# Environmental niche unfilling but limited options for range expansion by active dispersion in an alien cavity-nesting wasp

**DOI:** 10.1186/s12898-018-0193-9

**Published:** 2018-09-20

**Authors:** Carlo Polidori, Marcella Nucifora, David Sánchez-Fernández

**Affiliations:** 1 Instituto de Ciencias Ambientales (ICAM), Universidad de Castilla-La Mancha, Avenida Carlos III, s/n, 45071 Toledo, Spain; 2 Dipartimento di Scienze della Vita e dell’Ambiente, Università degli Studi di Cagliari, Via Fiorelli 1, 09126 Cagliari, Italy

**Keywords:** *Isodontia mexicana*, Niche unfilling, Biological invasion, BIOCLIM, Wood trade, Sphecidae

## Abstract

**Background:**

Predicting the patterns of range expansion of alien species is central to develop effective strategies for managing potential biological invasions. Here, we present a study on the potential distribution of the American cavity-nesting, Orthoptera-hunting and solitary wasp, *Isodontia mexicana* (Hymenoptera: Sphecidae), which was first detected as alien species in France in 1960 and now is present in many European countries. After having updated its current distribution, we estimated the environmental space (based on bioclimatic data and altitude) occupied by the species and subsequently predicted its environmental potential distribution under both present and future climatic conditions at global scale.

**Results:**

The wasp lives in low-altitude areas of the Northern hemisphere with moderate temperatures and precipitation. The environmental space occupied in the invaded area is practically just a subset (42%) of that occupied in the native area, showing a process of environmental niche unfilling (i.e. the species only partially fills its environmental niche in the invaded range). Besides, *I. mexicana* could also live in other temperate areas, mainly in the Southern hemisphere, particularly close to the coasts. However, geographic (oceans) and/or climatic (tropical areas, mountain chains) barriers would prevent the species to reach these potential areas unless through human trade activity. The species could thus only reach, by active dispersion, the remaining invadable areas of Europe. Estimations for the future (2050 and 2070) predict an expansion through active dispersion towards North in the native range and towards North and East in the invaded range, but future conditions would not break down the current climatic barriers in the Southern hemisphere.

**Conclusions:**

*Isodontia mexicana* has not shifted its environmental niche in the invaded area. It could still occupy some new areas by active dispersion, but confined to Europe. The conspicuous niche unfilling shown by this wasp species could reflect the likely single introduction in Europe just a few decades ago. Furthermore, results stay in line with other studies that found niche unfilling rather than niche expansion in insects.

**Electronic supplementary material:**

The online version of this article (10.1186/s12898-018-0193-9) contains supplementary material, which is available to authorized users.

## Background

Biological invasions are one of the most evident consequences of global change and a significant threat to native biodiversity [1–3]. Alien species are those species introduced outside its native range, either deliberately (e.g. as a part of pest biological control plans) or accidentally (e.g. through trade routes) [4], and that have been able to survive in the new habitats [5, 6]. Under certain conditions, some of these species may become invasive (i.e. they have a high growth rate, a fast range expansion, and greatly impact on the native biological communities) [7–9]. The number of invasion events by alien species is rapidly increasing worldwide [10, 11]. About 12,000 alien species are known to have been introduced to Europe, with about 40 of them of particular concern for their negative effects on the environment, thus calling for the development of effective strategies for managing biological invasions on the continent [12].

Given these observations, one important concern is to be able to estimate how far any invasive species could spread, thus allowing one to predict the patterns of range expansion [13, 14]. For this purpose, species distribution modelling has been widely used to forecast the potential distribution of a wide range of invasive species [15–22]. Because of the link between abiotic factors and physiological limits for survival of a species, factors such as the climate are commonly incorporated in species distribution modelling [20, 23, 24]. This is particularly important when assuming that the invasive species has already reached all suitable places and is absent from all unsuitable sites (i.e., the equilibrium assumption) and that the species’ ecological niche is stable in space and time (niche conservatism: the species does not shift to new environmental conditions after invasion) [25]. Furthermore, niche conservatism, which can be detected by large overlap between the niches of native and invaded areas, is not the only ecological pattern that a species may present after an introduction [26]. Indeed, when the niche of the invaded area is only a sub-space of the niche of the native area, the species shows niche unfilling [27]. On the other hand, when a new environmental space is occupied in the invaded range, the species is experiencing a niche shift or niche expansion [27, 28].

In this study, we focus on the predatory solitary wasp, *Isodontia mexicana* (de Saussure) (Sphecidae), a Nearctic species accidentally introduced into Europe probably about 60–70 years ago and first discovered in Hérault (southern France) in 1960 [29]. Since then, it has spread into many European countries [30]. *Isodontia mexicana* females nest in existing tunnels in wood and are specialized predators which use tree crickets (Gryllidae: Oecanthinae) and katydids (Tettigoniidae) to feed the brood [31–33]. The species probably reached Europe much likely as larvae or pupae present into woody objects transported by trade activity from the USA [30]. Kelner-Pillaut [29] even hypothesized that *I*. *mexicana* may have been introduced in Europe during World War II together with supplies for US military troops in 1944. For this species, no attempts have been previously made to predict the areas that could be still potentially invaded, both under the current and future climatic scenarios.

There are several reasons which makes *I. mexicana* an interesting species for such type of analyses. First, while some studies exist on the potential distribution of invasive social hymenopterans [34–37], there is just one single study to date on an invasive solitary bee [38] and no studies on any invasive aculeate solitary wasp. Thus, the information on this species may provide data for future analyses on the effect of social behaviour on potential spread after invasion. Second, a recent review [39] showed that niche conservatism is rare in invasive insect species, while many cases of niche unfilling and some cases of niche expansion have been found. Thus, data on this species may provide or not support for this general trend and may help testing hypotheses about which factors may have an influence on the occurrence of these alternative patterns. For example, niche unfilling seems more likely in case of single and recent introductions, compared to species with ancient colonization history and/or multiple introductions in different locations [40]. In our analysis, we refer specifically to the “environmental potential distribution”, which is the geographical representation of the environmental niche, i.e. the environmental space based on climate and altitude. We focus on environmental niche because available data on biotic determinants does not point towards limitations for this wasp in finding resources in invaded areas. For example, wasps were observed to often prey upon the same orthopteran subfamilies in both native and invaded areas, and almost all of the genera hunted in the native areas also occur in the invaded areas [31–33, 41–43]. Furthermore, the wasp was observed to nest in cavities of different wood types, including white pine and *Phragmites* reeds [31, 32, 44]. Third, although its impact on the local fauna has not been studied so far, *I. mexicana* may be exerting a competition pressure on the other two species of this genus native to Europe, *I. splendidula* (A. Costa) and *I. paludosa* (Rossi). Indeed, the three species largely use similar nesting and prey resources, and have a similar European distribution, sometimes even perfectly overlapping their ranges within countries (as in the case of France [41]).

Concretely, we aim to (a) update the current global distribution of the species, (b) estimate the environmental space (based on bioclimatic data and altitude) occupied by the species, (c) identify areas of the world that could potentially be invaded the species under the current environmental situation, and (d) identify the potential distribution under future scenarios of climate change.

## Methods

### Updating geographical distribution of *I. mexicana*

The distribution of *I. mexicana* was updated through the inspection of published sources (articles, books, official reports), data retrieved from GBIF (Global Biodiversity Information Facility) (www.gbif.org), confirmed observations available in entomological websites, and by adding unpublished observations recorded by one of the authors (*C. Polidori*) during trap-nest sampling in the field (a common method to collect cavity-nesting wasps) [e.g. 31, 45] carried out in Italy from 2008 to 2010 (Additional file 1). The complete dataset used in this study may potentially lack additional records, since, e.g., Museums collections have been not inspected. For example, the Western coast of the USA may be underrepresented in our dataset, and few European localities may have been not detected. Some publications may also have been overlooked, particularly if records of this wasp species are within large monographies on regional entomofauna. However, most part of the native range and almost the whole invaded range (i.e. all European countries recorded to date) seems to be covered, so that we think that possible records that have been not included would not affect our results.

Each distribution record was associated to its geographical attributes (latitude, longitude and altitude), retrieved directly from the data source or by searching the locality on Google Earth 6.2 (https://www.google.com/intl/en/earth). A database with all these records at a spatial resolution (grid cell size) of 0.4° was created. Each record was associated with a binary code identifying if it refers to either the native or the invaded area (Additional file 1). A total of 211 georeferenced points (129 native and 82 invaded) were obtained (Additional file 1).

### Estimating the environmental niche of *I. mexicana*

To estimate the abiotic environmental niche of *I. mexicana*, we used 20 environmental variables (19 bioclimatic variables plus altitude) associated to the occurrence points of the native and the invaded areas. We obtained the layers from Worldclim 1.4 (http://www.worldclim.org), with the same spatial resolution of the occurrence data (0.4°); these layers present data on altitude and seasonality trends, average and extreme values of temperatures and precipitation over the period 1950–2000 (Table 1).

**Table 1:** The 20 environmental (climate and altitude) variables retrieved from Worldclim and used in the study to build the environmental niche of *I. mexicana*

Worldclim code	Definition	Factor 1	Factor 2	Factor 3
BIO1	Annual mean temperature	0.935	0.325	− 0.102
BIO2	Mean diurnal range [mean of monthly (max temp − min temp)]	0.327	0.724	0.093
BIO3	Isothermality (BIO2/BIO7) (* 100)	0.911	0.047	0.132
BIO4	Temperature seasonality (standard deviation * 100)	− 0.900	0.019	− 0.138
BIO5	Max temperature of warmest month	0.734	0.561	− 0.249
BIO6	Min temperature of coldest month	*0.962*	0.161	− 0.054
BIO7	Temperature annual range (BIO5–BIO6)	− 0.843	0.187	− 0.108
BIO8	Mean temperature of wettest quarter	0.732	0.399	− 0.151
BIO9	Mean temperature of driest quarter	0.893	0.248	− 0.093
BIO10	Mean temperature of warmest quarter	0.797	0.490	− 0.251
BIO11	Mean temperature of coldest quarter	0.959	0.217	− 0.028
BIO12	Annual precipitation	0.669	− 0.699	0.096
BIO13	Precipitation of wettest month	0.701	− 0.464	0.326
BIO14	Precipitation of driest month	0.300	− 0.805	− 0.290
BIO15	Precipitation seasonality (coefficient of variation)	0.257	0.606	0.505
BIO16	Precipitation of wettest quarter	0.700	− 0.501	0.307
BIO17	Precipitation of driest quarter	0.334	*− 0.813*	− 0.277
BIO18	Precipitation of warmest quarter	0.469	− 0.598	0.214
BIO19	Precipitation of coldest quarter	0.487	− 0.630	− 0.133
ALTITUDE	–	− 0.146	− 0.009	*0.737*

The R^2^ of the correlations between the 20 variables and first three factors of the PCA obtained from these variables are shown (values in italic represent the variables most likely to explain the PCA factors)

Because of the autocorrelations among many of the 20 environmental variables and in the absence of any clear indication on which of them could be more important for the niche of *I. mexicana*, we performed a Principal Component Analysis (PCA) to reduce the number of variables to few ones that are not autocorrelated (the PCA factors). We selected the three first PCA factors (which explained the 81% of the total variance) and then identified the variable that most strongly correlated with these three factors (Table 1). These variables were minimum temperature of the coldest month (with F1), precipitation of driest quarter (with F2), and altitude (with F3). Values of these three variables were used to plot the global environmental space and to map localities from both the native range and the invaded range where *I. mexicana* has been recorded. We thus obtained a representation of the environmental space occupied by the species within the global one, as well as how much the environmental niches estimated from the native and invaded range overlap. This allowed ascertain if the species is experiencing niche conservatism, niche unfilling or niche expansion in the invaded area [26–28].

### Potential distribution of *I. mexicana* under current climate conditions

We estimated the global potential distribution of *I. mexicana* (i.e. the geographic area in which the abiotic environment is suitable to live; see [46]) by projecting the environmental niche built from first three PCA factors on the geographical space. This analysis was carried out through multidimensional envelope procedure (MDE), i.e. by using the minimum and maximum values of the variables extracted from the PCA factors [47]. Assuming that presence localities reflect a subset of the suitable conditions under which a species can survive, MDE is an approach directed at maximizing the capacity to represent geographically the potential distribution of species when they are only based on distributional data [46, 48, 49], so is being recommended as a conservative approach in biological invasions studies [46, 50].

To obtain continuous values for environmental suitability within the potential distribution of *I. mexicana*, we calculated the Mahalanobis distance (a measure of multidimensional non-Euclidean distance) between each one of the grid cells and the centroid of the hyper-volume of the selected variables. This procedure has been widely employed in spatial ecology [e.g. 22, 51, 52] and is commonly used to estimate the degree of suitability of the areas within the species potential distribution [53]. This analysis was carried out in Statistica 8.0 (StatSoft 2008).

### Potential distribution of *I. mexicana* under future climate conditions

The extreme values found above on minimum temperature of the coldest month and precipitation of driest quarter (as Altitude does not change with the climatic change) were projected with respect to future climate scenarios, to estimate the potential dynamics of invasion risk areas through time (i.e., combining current and future model outputs). Effects of climate change on the potential distribution were predicted considering the IPPC5 climate projections using the CCSM4 global climate model for 2050 (average for 2041–2060) and 2070 (average for 2061–2080), and one of the four representative concentration pathways (RCPs). The RCPs are consistent with a wide range of possible changes in future anthropogenic (i.e., human) greenhouse gas (GHG) emissions, and aim to represent their atmospheric concentrations. To be conservative within a framework of biological invasion, we considered the extreme scenario RCP 8.5, which assumes that global annual GHG emissions (measured in CO_2_-equivalents) will continue to rise throughout the twenty-first century. The layers with the future climatic data were obtained from Worldclim 1.4. (http://www.worldclim.org) [54], with the same spatial resolution of the occurrence data (0.4°).

## Results

**Fig. 1:**
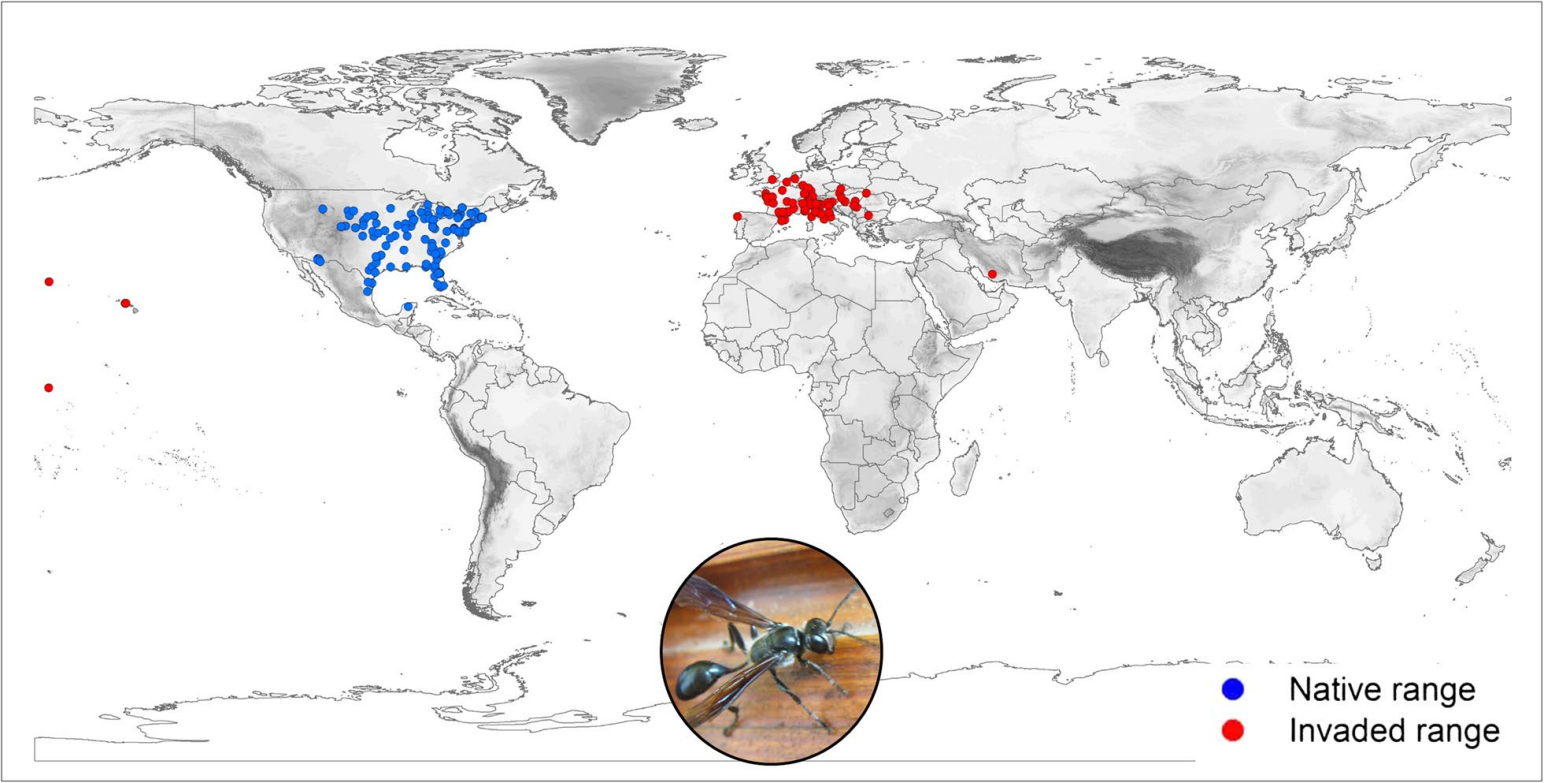
Actual distribution of *I. mexicana*, based on published and unpublished records (see Additional file 1). Occurrence records are in blue for the native areas and in red for the invaded areas

Currently, *I. mexicana* lives exclusively in the Northern hemisphere. The native range is confined to North America and extends from the USA (in the central and Eastern parts of the country) to Mexico (particularly its northern part) (see Fig. 1). With the exception of four occurrence records in Pacific islands [two in Hawaii (USA), one in Midway Atoll (USA) and one in Howland Island (USA)] and one record in Iran, the invaded range is confined to Europe, where the wasp occurs in many countries, particularly in France and Italy (and mostly in their Mediterranean areas), but also Switzerland, Austria, Germany and Serbia, among others (Additional file 2). The easternmost occurrence point in Europe is located in Ukraine, the northernmost record in southern England, the southernmost record in central Italy and the westernmost record is in northern Spain (Fig. 1).

**Fig. 2:**
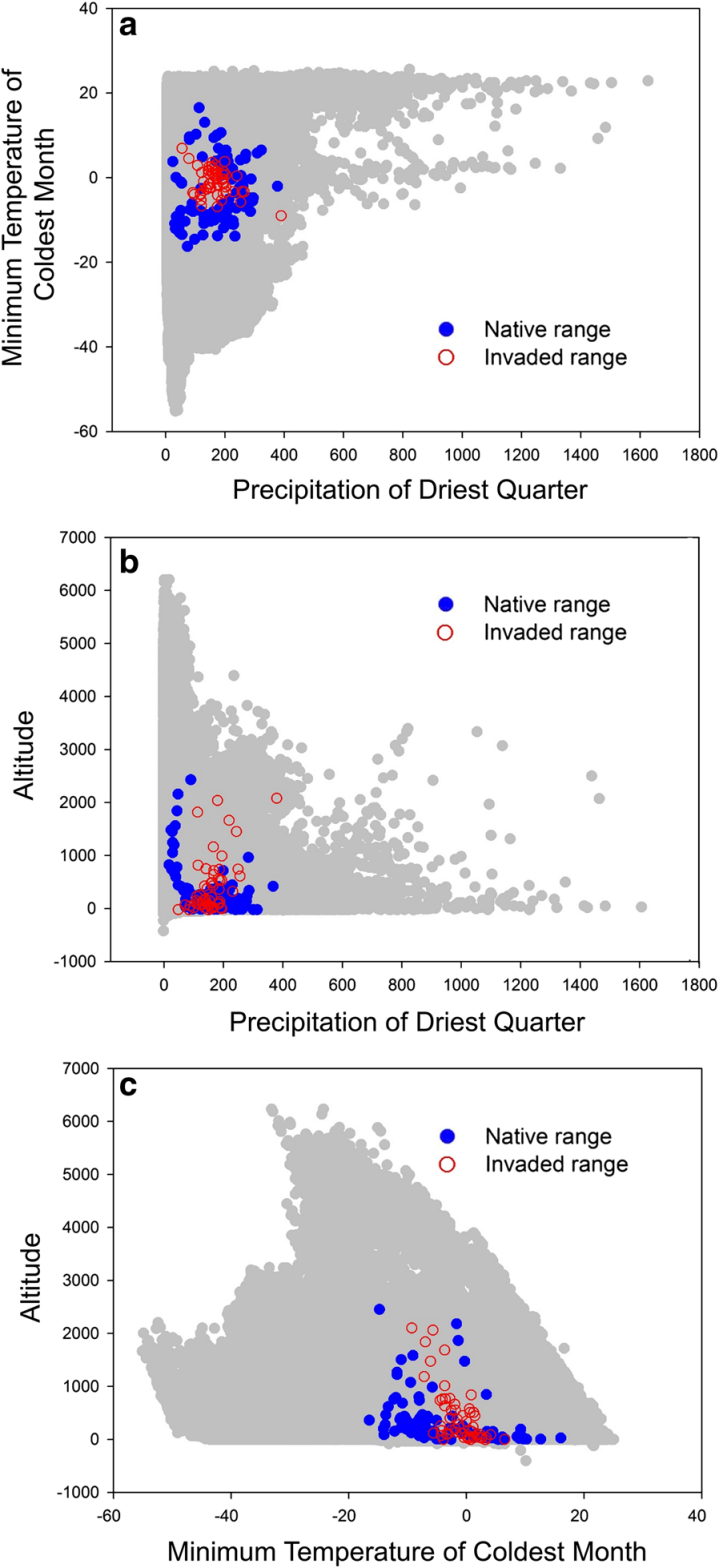
A Representation of the occupied environmental niche with plots showing the relationships among the variables that more strongly correlated with the three first principal factors obtained from a PCA built on 19 climate variables retrieved from Worldclim and altitude (Table 1).** a** Precipitation of Driest Quarter vs. Minimum Temperature of Coldest Month;** b** Precipitation of Driest Quarter vs. Altitude;** c** Minimum Temperature of Coldest Month vs. Altitude. Points in grey represent the global environmental conditions, blue points represent the occurrence records for the native areas, and red (empty) circles represents the occurrence records for the invaded areas. Temperature is in  °C, precipitation is in mm, and altitude is in m

By plotting the variables most correlated with the PCA factors obtained from the 20 environmental variables, *I. mexicana* showed a clear tendency to occupy areas with moderate values of temperature during winter (Minimum Temperature of the Coldest Month between − 16.6 and 15.9 °C, median value: − 3.2 °C), with few precipitation amounts in the driest period of the year (values for the driest quarter between 3 and 381 mm, median value: 167 mm), and low altitudes (between 0 and 2455 m asl, median value: 203 m) (Fig. 2). This is true considering both data from native and invaded areas, though in the latter the Minimum Temperature of the Coldest Month had a smaller range (− 10 °C to 10 °C). The environmental space occupied in the invaded areas almost completely falls within that occupied in the native area (95%). This 5% of the niche occupied in the invaded area which is not included in the niche occupied in the native area corresponded to a slightly warmer climate in winter. However, the environmental space occupied in the invaded areas does not completely overlap with the latter, showing some degree of niche unfilling (Fig. 2). In particular, the 42% of the native niche is occupied in the invaded areas by the wasp (i.e. 58% of the native niche is not filled) (Fig. 2).

**Fig. 3:**
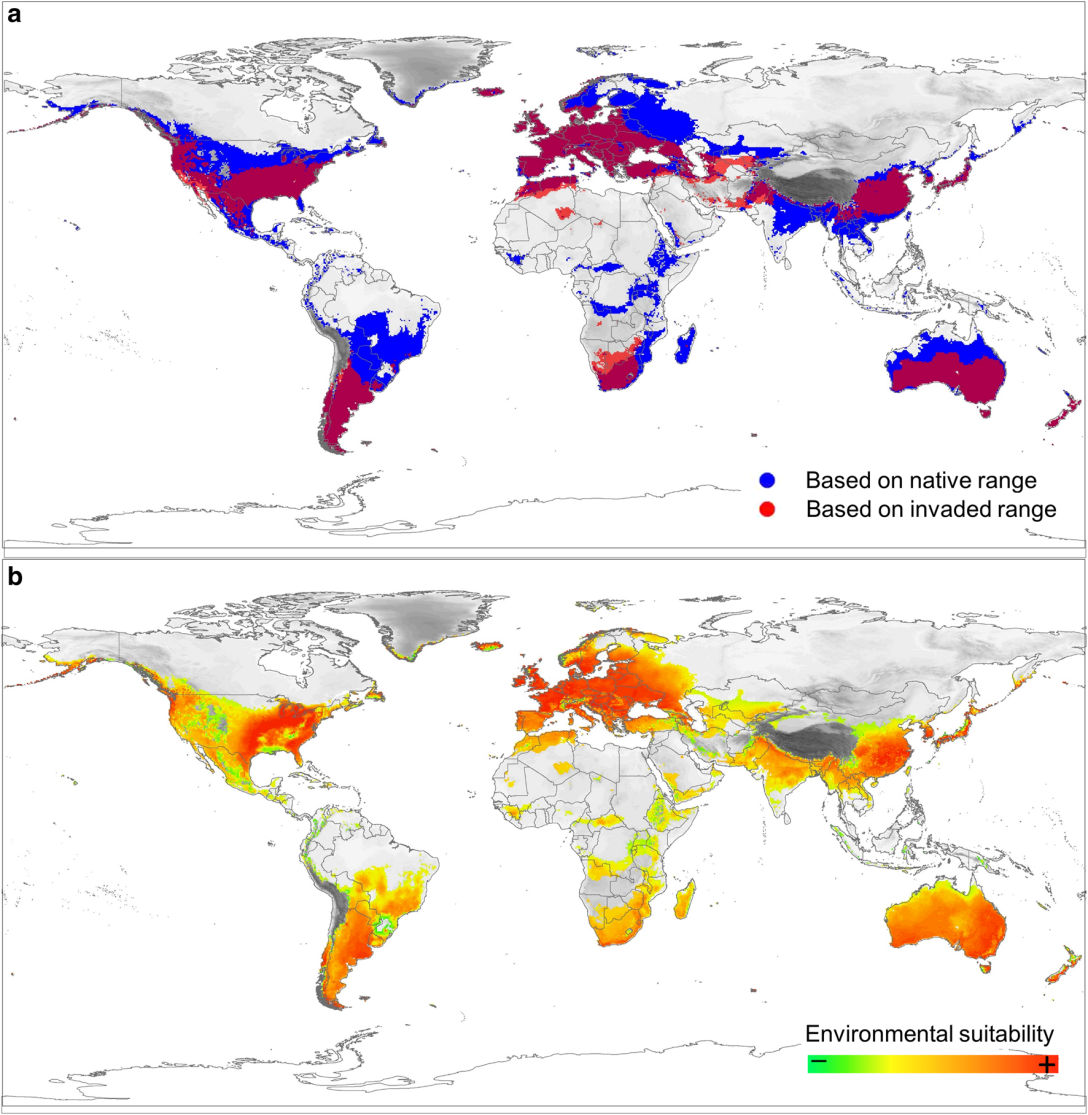
**a** Potential distribution of *I. mexicana*, based on the multidimensional envelope procedure (MDE), using data from the native range (in blue) and data from the invaded range (in red) (red grid points were made sufficiently transparent to highlight areas of overlap with blue grid points); **b** potential distribution of *I. mexicana* highlighting the degree if suitability (calculated through Mahalanobis distance method) for the species survival (increasing from green to red)

The potential distribution of *I. mexicana* obtained with MDE using all records (both from the native and invaded range) matches perfectly with the obtained using only the native records. Thus, the potential distribution based on the invaded range is a sub-set of those based on the native range (Fig. 3a). Based solely on the abiotic variable we investigated, the model predicts that the wasp species could establish itself in most of the remaining, still not occupied, parts of Europe, as well as some Asian countries (particularly the temperate/subtropical areas), and in different areas of the Southern hemisphere, including large portions of southern South America, Africa and Australia (Fig. 3a). On the other hand, although the wasp can be quite abundant in some of the Northern areas of the USA with very cold winters (Fig. 1), the wasp species would not be able to survive, at least based on our method, in the extremely cold conditions of the northernmost parts of North America and Asia, as well as in warm and tropical conditions of the northern parts of South America, central Africa and large Asian islands such as Indonesia, Philippines and Malaysia (Fig. 3a).

Within the areas that could be potentially invaded, those with higher levels of environmental suitability for the species are located in the eastern North America, most of Europe, the coastal areas of northern Patagonia, North Africa, South Africa, Southern Australia and Southern China (Fig. 3b). Desert or semi-desert areas (e.g. North to central Africa, tropical areas (e.g. Northern part of South America) and the lowland areas associated with large mountain chains (e.g. Andes, Himalaya) clearly showed the lowest degree of environmental suitability for the species (Fig. 3b).

**Fig. 4:**
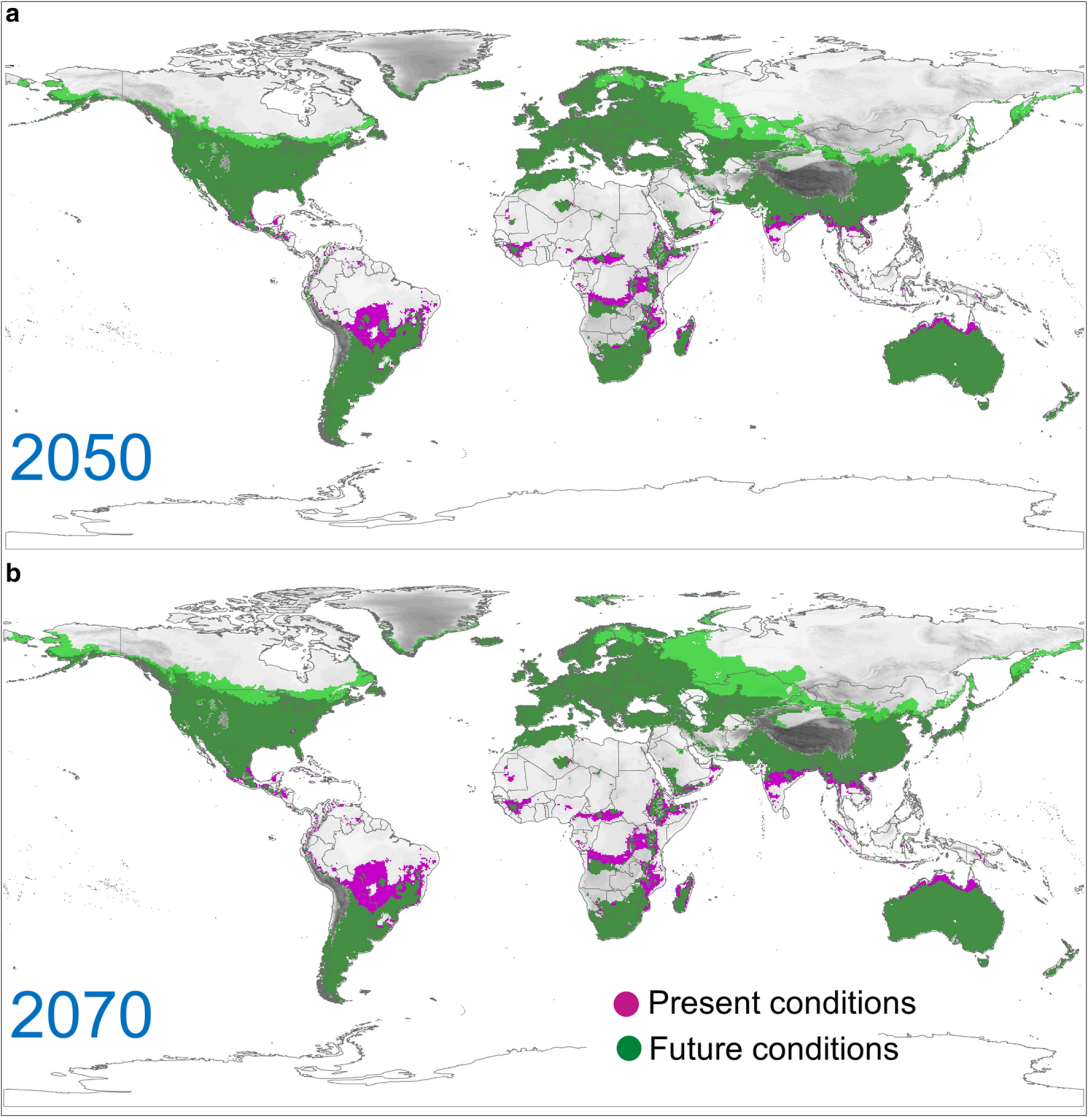
Estimate of the potential dynamics of invasion risk areas through time (i.e., combining current and future model outputs), based on the projections of the extreme values of Minimum Temperature of the Coldest Month and Precipitation of Driest Quarter (IPPC5 climate projections using the CCSM4 global climate model). Violet grid points: potential distribution under current conditions; Green grid points: potential distribution under future conditions. Green grid points were made sufficiently transparent to highlight areas of overlap with violet grid points. **a** Projection for 2050 under the RCP 8.5 concentration pathway; **b** projection for 2070 and RCP 8.5 concentration pathway

Our analysis of potential distribution of *I. mexicana* suggests that, under climate change, the species may also further spread towards North in the native range and towards North and East in the invaded range (Fig. 4), i.e. areas outside its potential range at present conditions. This prediction was similar for both climate scenarios (2050 and 2070), with just a further small expansion that may occur for the latter period. However, future conditions would eliminate the current climatically unsuitable areas in the Southern hemisphere (Fig. 4).

## Discussion

### Current distribution and potential spread based on environmental niche

Despite several recent papers reporting new occurrence records of *I. mexicana* have discussed the general distribution of the species in its invaded range [e.g. 55, 56], our study is the first one to pool together the available data and thus updated the current distribution of this wasp.

Based on the inspected sources for which the date of the species observation was available, it seems that *I. mexicana* could have extended its range within Europe in a roughly radial fashion. First, it established in France, then it moved to Italy, and subsequently, probably in parallel, towards East, towards West, and towards North. The most recent new records for the species (2017) come from the UK, Bulgaria and Galicia, thus accordingly to this radial spread (see Additional file 2). Today, the species occurs in at least 17 European countries. Thus, even if quite slowly, the wasp species is continuing occupying new areas within Europe, and, based on our estimations, it could likely occupy most of Europe in the future, where environmental conditions are highly suitable for the species. Mediterranean areas such as Southern Italy, Southern France and Greece are very suitable, in agreement with the establishment pattern observed for most of the 300 species of alien Hymenoptera today occurring in Europe [57]. Furthermore, our study predicts a high environmental suitability in coastal areas, which agrees with a recent study at global scale, which demonstrated how these areas are already those, together with islands, most affected by biological invasions and those harbouring the highest richness of already established alien species [58].

Besides Europe, *I. mexicana* is predicted to find suitable conditions to live (though at different degrees of suitability) also in different areas of other continents, including many in the Southern hemisphere. However, geographical barriers (seas, oceans) or large environmentally unsuitable areas would make impossible (the former) or very difficult (the latter) for the species to reach Australia, Africa, Patagonia and Eastern Asia by natural, active dispersion. *Isodontia mexicana* could thus experience a further range expansion within Europe by active dispersion, but it would reach other continents only through human trade activity.

An interesting result of our study is that the niche estimated from the invasive range is a sub-component of the niche estimated from the native range, pointing to unfilled niche in the invaded area. This pattern indicates that the distribution of this species is not in equilibrium with the environmental conditions, since the species has not occupied yet all the environmental space in which it can survive. While niche conservatism limits the uncertainty associated with niche shifts from native to invaded areas [59], niche unfilling is less problematic than niche expansion for distribution predictions. In fact, as for niche conservatism, niche unfilling also implies that the species would not occupy new areas with a climate different from that of the native areas. In any case, even though niche conservatism seems to be an a priori assumption for predicting potential invadable areas [46], observation of niche changes are increasingly reported [17, 60, 61]. Niche unfilling seems to be common for species at the first phases of invasion, which are dependent of the dispersal capability of the species, and more likely in case of single introductions [40]. This situation seems compatible with the known invasion process of *I. mexicana*, which likely arrived to Europe through a single introduction just a few decades ago. In agreement with this point, niche unfilling seems quite conspicuous for this wasp species (58% of the niche is unoccupied). When comparing with other invasive insect species, at first one notes that niche conservatism is rare and niche unfilling and niche expansion common [39]. However, the degree of unfilling and expansion is extremely variable. Hill et al. [39] found indeed that only six out of 22 species analysed have about half of the niche unfilled (based on climatic variables), while in most cases the niche was almost completely filled, and in only four cases an almost complete unfilling appeared. Furthermore, only five of the insect species analysed show conspicuous niche expansion (> 30%), which agrees with our finding of very weak niche shift (5%). Hill et al. [39] revealed also that alien insect species tend to invade areas with levels of human disturbance similar to their native range. At least, the human population density (one of the factor used in Hill et al. [39]) of most of the native and invaded areas of *I. mexicana* seem quite comparable (e.g. roughly 100–200 persons/km^2^ in Italy and France (the non-native countries more occupied by the wasp), and in the eastern USA states (the ones more occupied by the wasp in the native range). Of course, areas of high population density are also those with more occurrence of entomologists, which could affect analyses on distribution and expansion track.

Contrary to our results on the solitary *I. mexicana*, in social Hymenoptera niche shift after invasion seems more common. Indeed, a variety of niche shift patterns were observed in the invasive bumblebee *Bombus terrestris* (L.), in the invasive wasps *Vespa velutina* Lepeletier and *Vespula germanica* (Fabricius) and in most of the invasive ants studied so far [36, 37, 39, 62, 63]. Interestingly, the only other investigated alien solitary aculeate, the bee *Anthidium manicatum* (L.), also showed some degree of niche shift, particularly in the southern part of its invaded range (South America) [38]. However, one notes that this niche shift appeared to be much more reduced in this species than in the social species studied so far [39]. Studying more solitary and social species is thus necessary to verify if niche shift is associated with sociality or other factors (e.g. generalism vs. specialization in food resource use) in invasive Hymenoptera.

Despite both niche expansion and niche unfilling result in a reduced overlap between native and invaded niches, these two main types of niche changes are driven by different processes. Indeed, niche unfilling might be the pure result of ongoing colonization and slow dispersal [21, 27, 40, 64] (that could apply to *I. mexicana* in Europe) or to the impossibility to reach new areas with the still-to-fill environmental conditions (that could apply to *I. mexicana* for the non-European suitable areas). Niche unfilling may potentially also precede further range expansion [39], although for *I. mexicana* this further expansion would be very limited (through active dispersion), following the considerations expressed above.

### Future potential distribution

Predictions of *I. mexicana* future potential range suggest climate change may enlarge the colonized areas. In general, both increased temperature and higher frequency of extreme climatic events (e.g. intense heat waves, hurricanes, floods) are expected to affect the establishment of new alien species in territories which are currently unoccupied [65, 66]. This seems particularly true for terrestrial arthropods, because of their ectothermic physiology and their often high dispersal rate, and because they are commonly dispersed by humans through trade activity [66]. In the case of *I. mexicana*, however, the expected range expansion in 2050 and in 2070 (under the RCP 8.5 scenario) would not allow the species to reach suitable areas in the Southern hemisphere by active dispersion, since the climatic barriers in South America would still persist, and the species is not expected to shift its environmental niche. Africa and Australia will still be impossible to reach by active dispersion because of sea barriers. Thus, the species would only be able to expand further through Northern Europe and Asia north to Himalaya. In addition, *I. mexicana* would not rapidly establish in new areas, given that, among other factors, the rapid tracking of climate change should be more facilitated by having many generations per year and high dispersal ability [66], which does not apply to this wasp species. On the other side, *I. mexicana* may be favoured in being mostly a low- to moderate-altitude species better suited to future warmer and drier climates. An increase in the extent of suitable areas to invade under climate change scenarios was predicted for other aculeate Hymenoptera [67, 68].

Range expansion of *I. mexicana* should not be limited by food resources, as long as prey taxa occur in adequate abundances of individuals. Indeed, Gryllidae and Tettigoniidae, the only two orthopteran families hunted in both native [32, 33, 69–72] and invaded [42–44, 73, 74] areas, have a cosmopolitan distribution (http://orthoptera.speciesfile.org/HomePage/Orthoptera/HomePage.aspx) and can be thus used as prey in Europe and in other continents. Many of the genera known to be used as prey also occur in many continents (Additional file 3). Furthermore, while certain genera (notably *Oecanthus*) shows the highest frequency as prey in both native and invaded areas (Additional file 3), a number of genera are used as prey only in the native area (because lacking in the invaded area) [32, 69–71], other genera are hunted apparently only in the invaded area despite also occurring in the native area, and one genus lacking in North America was even added as a new prey in the invaded area [75] (Additional file 3). This suggests that the wasp is able to enlarge its prey spectrum in the invaded area. The occurrence of the two native European *Isodontia* apparently does not prevent the range expansion of *I. mexicana*, which, because of the similarity in prey spectrum (Gryllidae and Tettigoniidae) and nesting substrate (woody-existing cavities) [41], could potentially compete with these native species, an aspect which was not investigated so far.

We are aware that our data set has limitations that could be not controlled here and may affect our results by underestimating the environmental conditions that the species is able to tolerate, and thus its potential distribution. For example, the analysis treats each record equally even though the wasp abundance may be very variable among sites, which may result in an overestimate of niche breadth and potential distribution [76, 77]. Furthermore, distribution data could also depend on the distribution of entomologists, which is typically uneven in large areas, such as North America, and this may also result in bias in the association of distribution with climatic variables.

## Conclusions

We predict that *I. mexicana* can spread further through Europe and, only through human trade activity, can reach and survive in new, still unoccupied continents in the Southern Hemisphere.

With the aim to prevent future invasions, we recommend to focus monitoring efforts in areas with suitable environmental conditions but not invaded yet, especially those with wood trade connections with areas already occupied by the species. To prevent further human-mediated dispersion of this species it will be important to pay attention to the trade routes involving the transportation of lumber and wood product that might harbour nests with living wasp larvae or pupae, particularly given the increase of the rate of merchandise transport across the globe after the 1950s through technical and logistic improvements [78, 79]. This will be particularly important for sea shipping, an important way of wood or woody objects trade, and an extremely successful way to involuntarily import alien species to new areas [80–82]. Officials which inspect imported wood products could be trained to recognize specific nest structures of potentially invasive species, such as the characteristic grass plugs that *I. mexicana* females add to the entrance of their nest tunnels [31, 32, 44].

## Additional files

**Additional file 1.** Georeferenced records of *Isodontia mexicana* in its native and invaded range.

**Additional file 2.** Georeferenced records of *Isodontia mexicana* in Europe (invaded range). The suspected year of arrival of the species, based on the year of the first detection, and the year of the last record, for each of the 17 invaded countries is shown.

**Additional file 3.** Graphical summary on the known prey use (17 orthopteran genera) by *Isodontia mexicana* in its native and invaded range. World maps show the rough distribution for each of the 17 genera (“+” indicates occurrence in a given continent; data obtained from GBIF and http://orthoptera.speciesfile.org/HomePage/Orthoptera/HomePage.aspx). Squares indicates the % of the records (*n* = 47) in which a given genus was found as prey, in both native (white half-square) and invaded (black half-square) range. Picture: *I. mexicana* female at nest.
