# Supplementary material for: Environmental niche unfilling but limited options for range expansion by active dispersion in an alien cavity-nesting wasp
Source: BMC Ecol. 2018 Sep 20;18:36. doi: 10.1186/s12898-018-0193-9 (PMC6148766; doi:10.1186/s12898-018-0193-9)
Supplement: Supplementary file 1 — Additional file 1. Georeferenced records of Isodontia mexicana in its native and invaded range. [file 12898_2018_193_MOESM1_ESM.pdf]

Additional file 1. Georeferenced records of *Isodontia mexicana* in its native and invaded range.

Observation	Latitude	Longitude	Native (0) or invaded (1) area	Source
1	-122.236111	37.482778	0	Bugguide (2010)
2	-110.993702	31.854251	0	Bohart and Menke (1963)
3	-110.975779	32.262393	0	Bohart and Menke (1963)
4	-110.737306	32.251742	0	Bohart and Menke (1963)
5	-110.4075	31.4889	0	GBIF 1062029364
6	-109.733566	44.316452	0	Bohart and Menke (1963)
7	-105.2727	40.0232	0	GBIF 1101329158
8	-105.2705	40.015	0	GBIF 1101329148
9	-104.2778	40.2392	0	GBIF 1101329132
10	-104.271	40.195	0	GBIF 1101329126
11	-103.66714	43.91665	0	Bohart and Menke (1963)
12	-103.2694	42.7457	0	GBIF 1101329133
13	-102.204	43.8595	0	GBIF 1136074203
14	-101.8008	39.7714	0	GBIF 1101329093
15	-100.017079	37.752798	0	Bohart and Menke (1963)
16	-99.897795	40.306258	0	Bohart and Menke (1963)
17	-99.885753	41.368901	0	Bohart and Menke (1963)
18	-99.602285	41.645085	0	Bohart and Menke (1963)
19	-98.884227	42.514066	0	Bohart and Menke (1963)
20	-98.789994	24.27018	0	Vanoye-Eligio et al. (2015)
21	-98.7667	26.5667	0	GBIF 1062029384
22	-98.493628	29.424122	0	Bohart and Menke (1963)
23	-97.755493	26.25181	0	Porter (1978)
24	-97.751101	30.289247	0	Bohart and Menke (1963)
25	-97.743061	30.267153	0	Bohart and Menke (1963)
26	-97.611424	38.840281	0	Bohart and Menke (1963)
27	-97.362324	42.698662	0	Bohart and Menke (1963)
28	-97.330053	37.687176	0	Bohart and Menke (1963)
29	-97.249497	42.71472	0	Bohart and Menke (1963)
30	-97.02532	28.86446	0	GBIF 1305156310
31	-96.84304	32.78899	0	GBIF 1272103370
32	-96.701949	31.244281	0	Bohart and Menke (1963)
33	-96.685198	40.825763	0	Bohart and Menke (1963)
34	-96.474916	32.463599	0	Bohart and Menke (1963)
35	-96.181663	38.403903	0	Bohart and Menke (1963)
36	-95.601067	33.138448	0	Bohart and Menke (1963)
37	-95.600618	33.138462	0	Bohart and Menke (1963)
38	-94.578567	39.099726	0	Bohart and Menke (1963)
39	-94.398547	35.385924	0	Bohart and Menke (1963)
40	-93.292299	37.208957	0	Bohart and Menke (1963)
41	-93.217376	30.226595	0	Bohart and Menke (1963)
42	-93.18289	44.46587	0	GBIF 1088903477

43	-92.200723	37.828652	0	Bohart and Menke (1963)
44	-91.433805	40.351038	0	McCravy et al. (2009)
45	-89.944257	34.311498	0	Bohart and Menke (1963)
46	-89.6562	39.57686	0	GBIF 1060918953
47	-89.588986	40.693649	0	Bohart and Menke (1963)
48	-89.330046	30.308808	0	Bohart and Menke (1963)
49	-89.21675	37.727273	0	Bohart and Menke (1963)
50	-88.899167	20.598611	0	Vanoye-Eligio et al. (2015)
51	-88.243383	40.11642	0	Bohart and Menke (1963)
52	-88.231481	43.011678	0	Bohart and Menke (1963)
53	-87.861153	41.120032	0	Bohart and Menke (1963)
54	-87.629798	41.878114	0	Bohart and Menke (1963)
55	-87.52744	41.68713	0	GBIF 1262945497
56	-85.771997	43.550297	0	Bohart and Menke (1963)
57	-85.000521	41.940326	0	Bohart and Menke (1963)
58	-84.8494	36.9908	0	GBIF 1136065932
59	-84.647633	30.188423	0	Manley et al. (1997)
60	-84.57547	30.9038	0	Bohart and Menke (1963)
61	-84.280733	30.438256	0	Bohart and Menke (1963)
62	-84.247212	43.615583	0	Bohart and Menke (1963)
63	-84.223903	45.357512	0	Bohart and Menke (1963)
64	-83.929395	42.607255	0	Bohart and Menke (1963)
65	-83.65966	41.957268	0	Bohart and Menke (1963)
66	-83.357567	33.951935	0	Bohart and Menke (1963)
67	-83.187658	35.967041	0	Bohart and Menke (1963)
68	-83.127766	35.168357	0	Bohart and Menke (1963)
69	-83.0167	39.9667	0	GBIF 1062029379
70	-83.0003	40.2667	0	GBIF 1062029302
71	-82.9027	30.3091	0	Campbell et al. (2017)
72	-82.837365	34.683438	0	Bohart and Menke (1963)
73	-82.8208	41.6542	0	GBIF 1062029340
74	-82.8208	41.6542	0	GBIF 1062029297
75	-82.4667	39.5167	0	GBIF 1062029310
76	-82.457178	27.950575	0	Bohart and Menke (1963)
77	-82.3383	29.824	0	Campbell et al. (2017)
78	-82.333179	30.666893	0	Bohart and Menke (1963)
79	-82.324826	29.651634	0	Bohart and Menke (1963)
80	-82.23485	33.35217	0	GBIF 1060919125
81	-82.1719	29.4043	0	Campbell et al. (2017)
82	-82.1433	29.407	0	Campbell et al. (2017)
83	-82.1165	28.9691	0	Campbell et al. (2017)
84	-81.904005	32.161581	0	Bohart and Menke (1963)
85	-81.7376	27.9514	0	Campbell et al. (2017)
86	-81.707536	25.939741	0	Bohart and Menke (1963)
87	-81.5026	28.7336	0	GBIF 1075093071
88	-81.269453	28.802861	0	Bohart and Menke (1963)

89	-81.245277	42.984923	0	Bohart and Menke (1963)
90	-81.2086	28.6633	0	GBIF 1045112169
91	-81.2069	28.5925	0	GBIF 1065069654
92	-81.1939	28.6044	0	GBIF 1045112146
93	-81.144	44.127	0	GBIF 1416184316
94	-81.034814	34.00071	0	Bohart and Menke (1963)
95	-81.025776	25.300301	0	Bohart and Menke (1963)
96	-80.855648	33.49182	0	Bohart and Menke (1963)
97	-80.357827	25.666034	0	Bohart and Menke (1963)
98	-80.224528	43.533155	0	O’Neill and O’Neill (2009)
99	-79.791975	36.072635	0	Bohart and Menke (1963)
100	-79.528143	43.92869	0	Entomoland (2003)
101	-79.45467	38.8635	0	GBIF 1136062331
102	-79.340686	43.716589	0	Entomoland (2003)
103	-78.878369	42.886447	0	Bohart and Menke (1963)
104	-77.8409	39.5188	0	GBIF 1136023725
105	-77.719993	39.641762	0	Bohart and Menke (1963)
106	-77.6303	39.3127	0	GBIF 1136052483
107	-76.8713	42.3756	0	GBIF 1136060347
108	-76.8088	42.0959	0	GBIF 1136060368
109	-76.711322	43.022657	0	O’Neill and O’Neill (2009)
110	-76.678	38.8993	0	GBIF 1136063475
111	-76.56272	43.048677	0	O’Neill and O’Neill (2009)
112	-76.5175	38.5361	0	GBIF 1136022364
113	-76.501881	42.443961	0	Bohart and Menke (1963)
114	-76.147424	43.048122	0	Bohart and Menke (1963)
115	-75.773	44.621	0	GBIF 1415613785
116	-75.165222	39.952584	0	Bohart and Menke (1963)
117	-75.0726	38.6813	0	GBIF 1136017819
118	-74.726579	39.927429	0	Bohart and Menke (1963)
119	-74.5775	40.6647	0	GBIF 1062029334
120	-74.171811	40.916765	0	Bohart and Menke (1963)
121	-74.005941	40.712784	0	Bohart and Menke (1963)
122	-73.675	43.2475	0	GBIF 1136000498
123	-73.538734	41.05343	0	Bohart and Menke (1963)
124	-72.518511	43.624244	0	Bohart and Menke (1963)
125	-71.973963	41.948431	0	Bohart and Menke (1963)
126	-71.05888	42.360082	0	Bohart and Menke (1963)
127	-71.00861	42.31722	0	GBIF 788917826
128	-70.92056	42.26944	0	GBIF 788917755
129	-70.87694	42.26389	0	GBIF 788917886
130	-8.6475	42.433611	1	Biodiversidad virtual (2017)
131	-1.677793	48.117266	1	Le Monde des insectes (2016)
132	-1.59173	47.234779	1	Le Monde des insectes (2016)
133	-1.553621	47.218371	1	Le Monde des insectes (2016)
134	-1.412569	47.080178	1	Le Monde des insectes (2016)

135	-1.154003	46.190717	1	Pagliano et al. (2000)
136	-0.126883	51.501719	1	Notton (2016)
137	-0.11281	47.266278	1	Le Monde des insectes (2016)
138	-0.089808	47.242846	1	Le Monde des insectes (2016)
139	0.153476	45.751996	1	Le Monde des insectes (2016)
140	1.444209	43.604652	1	Pagliano et al. (2000)
141	1.851472	42.937653	1	Le Monde des insectes (2016)
142	2.176944	41.3825	1	Biodiversidad virtual (2017)
143	2.31937	43.57193	1	GBIF 1136848330
144	2.352222	48.856614	1	Le Monde des insectes (2016)
145	2.381362	43.072467	1	Herbrechet (2010)
146	2.930529	41.722493	1	Pagliano et al. (2000)
147	3.258363	43.591236	1	Herbrechet (2010)
148	3.334601	50.920201	1	Natuur.forum Vlaanderen (2010)
149	3.425488	46.131859	1	Le Monde des insectes (2016)
150	3.476811	43.308802	1	Pagliano et al. (2000)
151	3.899178	43.701947	1	Pagliano et al. (2000)
152	4.835659	45.764043	1	Le Monde des insectes (2016)
153	5.096869	44.500121	1	Pagliano et al. (2000)
154	5.352881	51.641348	1	Smit and Wijngaard (2010)
155	7.23	45.716	1	GBIF 886476619
156	7.315963	50.134823	1	Tischendorf et al. (2011)
157	7.65	48.08	1	GBIF 886477218
158	7.67	48.08	1	GBIF 886476650
159	7.686856	45.070312	1	Pagliano et al. (2000)
160	7.776057	43.815967	1	Pagliano et al. (2000)
161	7.819671	44.395343	1	Pagliano et al. (2000)
162	8.013794	43.900541	1	Pagliano et al. (2000)
163	8.46604	49.487459	1	Tischendorf et al. (2011)
164	8.50761	49.49672	1	GBIF 1338867790
165	8.6259	46.897037	1	Vernier (1995)
166	8.672288	49.394667	1	Tischendorf et al. (2011)
167	8.744338	46.186706	1	Vernier (1995)
168	8.754057	45.599135	1	Polidori et al., unpublished data
169	8.884985	45.465756	1	Polidori et al., unpublished data
170	8.970465	46.358001	1	Vernier (1995)
171	8.993333	45.287198	1	Polidori et al., unpublished data
172	9.024611	45.8367	1	Pagliano et al. (2000)
173	9.028129	45.258157	1	Polidori et al., unpublished data
174	9.057645	48.521636	1	Tischendorf et al. (2011)
175	9.360155	48.657519	1	Zettel (2003)
176	9.4	45.416667	1	Naturalmediterraneo (2017)
177	9.5078	42.445813	1	Pagliano et al. (2000)
178	9.74379	47.4124	1	Ercit (2014)

179	10.010019	45.891385	1	Polidori et al., unpublished data
180	10.310567	43.548473	1	Pagliano et al. (2000)
181	10.362439	43.722357	1	Pagliano et al. (2000)
182	10.401689	43.722839	1	Pagliano et al. (2000)
183	10.43	45.65	1	GBIF 886477012
184	10.83	45.56	1	GBIF 886476840
185	11.330757	43.318809	1	Pagliano et al. (2000)
186	11.342616	44.494887	1	Pagliano et al. (2000)
187	11.535421	45.545479	1	Pagliano et al. (2000)
188	11.787247	43.098694	1	Pagliano et al. (2000)
189	11.876761	45.406435	1	Pagliano et al. (2000)
190	12.496366	41.902784	1	Pagliano et al. (2000)
191	13.48	45.85	1	GBIF 11751169
192	13.63	45.08	1	GBIF 11751218
193	13.72	43.45	1	GBIF 11751219
194	14.163585	42.347886	1	Pagliano et al. (2000)
195	14.505314	46.05563	1	Ćetkov et al. (2012)
196	16.218506	48.126782	1	Zettel (2003)
197	16.616667	49.2	1	Říha (2017)
198	16.674773	46.98796	1	Ćetkov et al. (2012)
199	17.345556	48.298056	1	Semelbauer (2015)
200	17.45426	46.201634	1	Ćetkov et al. (2012)
201	17.644966	46.144818	1	Ćetkov et al. (2012)
202	19.709344	45.157061	1	Ćetkov et al. (2012)
203	20.141425	46.25301	1	Ćetković et al. (2012)
204	20.448922	44.786568	1	Ćetković et al. (2012)
205	22.856098	48.175027	1	Fateryga et al. (2014)
206	23.323638	42.697556	1	Gradinarov (2017)
207	34.579779	45.857051	1	Fallahzadeh et al. (2009)
208	-176.618274	0.811322	1	Vanoye-Eligio et al. (2015)
209	-157.94772	21.334	1	GBIF 1060919059
210	-157.7615	21.37337	1	GBIF 1060919117
211	-177.366667	28.216667	1	Nishida et al. (2002)

Sources:

Biodiversidad virtual (2017) http://www.biodiversidadvirtual.org/insectarium/Isodontia-mexicana-(Saussure-1867)-img913537.html. Accessed May 2018.

Biodiversidad virtual (2017) http://www.biodiversidadvirtual.org/insectarium/Isodontia-mexicana-(Saussure-1867)-img914323.html. Accessed May 2018.

Bohart RM, Menke AS (1963) A reclassification of the Sphecinae, with a revision of the nearctic species of the tribes Scleiphronini and Sphecini (Hymenoptera:Sphecidae). Univ. Calif. Publ. Entomol. 30: 91-182.

Bugguide (2010) https://bugguide.net/node/view/402190/bgimage. Accessed May 2018.

Campbell J, Smithers C, Irvin A, Kimmel C, Stanley-Stahr C, Daniels J, Ellis J (2017) Trap Nesting Wasps and Bees in Agriculture: A Comparison of Sown Wildflower and Fallow Plots in Florida. Insects 8: 4, 107.

Ćetković A, Čubrilović B, Plećaš M, Popovć A, Saić D, Stanisavlje L (2012) First records of the invasive American wasp Isodontia mexicana (Hymenoptera: Sphecidae) in Serbia. Acta entomologica serbica 17(1/2): 63-72.

Entomoland (2003) http://denbourge.free.fr/Insectes_hymenoptera_apocrita_Sphecidae_Isodontia_mexicana.htm. Accessed February 2017.

Ercit K (2014) Size and sex of cricket prey predict capture by a sphecid wasp. Ecol. Entomol. 39:195–202.

Fallahzadeh M, Ostovan H, Saghaei N (2009) A contribution to the fauna of Sphecidae and Crabronidae(Hymenoptera) in FarsProvince, Iran. Plant Protection Journal, 1: 234–248.

Fateryga VA, Protsenko Yu V, Zhidkov V Yu (2014) *Isodontia mexicana* (Hymenoptera, Sphecidae), a new invasive wasp species in the fauna of Ukraine reared from trap-nests in the Crimea. *Vestnik zoologii*, 48: 185–188

GBIF (Global Biodiversity Information Facility) (www.gbif.org). Accessed February 2017.

Gradinarov D (2017) First records of the American wasps *Sceliphron caementarium* (Drury, 1770) and *Isodontia mexicana* (de Saussure, 1867) (Hymenoptera: Sphecidae) from Bulgaria. ZooNotes, 2017: 1-4.

Herbrecht F (2010) Découvertes récentes d'*Isodontia mexicana* (Saussure, 1867) dans le Nord-Ouest de la France (Hymenoptera, Sphecidae). Invertébrés Armoricains, 6: 45-46.

Le Monde des insectes (2016) https://www.insecte.org/forum/viewtopic.php?t=620&start=40. Accessed February 2017.

Manley DG, Carithers TP (1998) A new host record for *Sphaeropthalma pensylvanivca pensylvanica* (Hymenoptera: Mutillidae). Entomological News 109:198–200.

McCravy KW, Bara JJ, Hessler SN, Luxmore LK, Stinebaker KS, Jenkins SE (2009) Abundance and diversity of thread-waisted wasps (Hymenoptera: Sphecidae: Sphecinae) at Alice L. Kibbe Life Science Station, Hancock County, IL, USA. Trans. Ill. State Acad. Sci., 102, 107–115.

Naturalmediterraneo (2017) https://www.naturamediterraneo.com/forum/topic.asp?TOPIC_ID=296892. Accessed May 2018.

Natuur.forum Vlaanderen (2010) http://www.natuur-forum.be/phpBB3/viewtopic.php?f=16&p=210101

Nishida, GM, Beardsley JW (2002) A review of the insects and related arthropods of Midway Atoll. Bishop Museum Occasional Papers 68: 25-69.

Notton DG (2017) Grass-Carrying Wasp, *Isodontia Mexicana* (De Saussure), genus and species new to Britain (Hymenoptera: Sphecidae). British Journal of Entomology and Natural History 29: 241–245.

O'Neill KM, O'Neill FJ (2009) Prey, Nest Associates, and Sex Ratios of *Isodontia mexicana* (Saussure) (Hymenoptera: Sphecidae) from Two Sites in New York State. Entomologica Americana 115(1): 90-94

Pagliano G, Scaramozzino PL, Strumia F (2000) Introduction and spread of four Aculeate Hymenopterain Italy, Sardinia and Corsica. *Hymenoptera. Evolution, Biodiversity and Biological Control* (ed. by A.D. Austin and M. Dowton), pp. 290–295, CSIRO Publishing, Collingwood, Australia.

Porter CC (1978) Ecological Notes on Lower Rio Grande Valley Sphecini. Florida Entomol. 61: 169-178.

Říha M (2017) Species Diversity and Habitat Preferences of Aculeata (Insecta: Hymenoptera) of Urban and Suburban Gardens in Brno-City (Czech Republic). Acta Universitatis Agriculturae et Silviculturae Mendelianae Brunensis, 65: 0171–0178.

Semelbauer M (2015) American wasp *Isodontia mexicana* (Hymenoptera: Sphecidae) in Slovakia. Entomofauna Carpathica, 27(2): 10-11.

Smit J, Wijngaard W (2010) *Isodontia mexicana*, een nieuwe langsteelgraafwesp voor Nederland (Hymenoptera:Sphecidae) // Nederlandse Faunistische Mededelingen, 67–72.

Tischendorf S, Frommer U, Flügel H-J (2011) Kommentierte Rote Liste der Grabwespen Hessens (Hymenoptera: Crabronidae, Ampulicidae, Sphecidae) – Artenliste, Verbreitung,Gefährdung. Hessische Ministerium fürUmwelt, Energie, Landwirtschaft und Verbraucherschutz, Wiesbaden.

Tussac H, Voisin J-F (1989) Observation sur la nidification *d'Isodontia mexicana* (SAUSSURE,1867) en France et en Espagne. Bulletinde laSociété entomologique de France 94:109-111.

Vanoye-Eligio M., Meléndez-Ramírez V, Ayala R, Navarro-Alberto J, Delfín-González H (2015) Avispas depredadoras de áreas naturales protegidas del estado de Yucatán, México. Revista Mexicana de Biodiversidad 86: 989–997.

Vernier R (1995) *Isodontia mexicana* (Sauss.), un Sphecini américain naturalisé en Suisse (Hymenoptera, Sphecidae). Band (Jahr): 68.

Westrich P (1998) Die Grabwespe *Isodontia mexicana* (Saussure, 1867) nun auch in Deutschland gefunden(Hymenoptera, Sphecidae). Entomologische Zeitschrift, 108(1): 24–25.

Zettel H (2003) *Isodontia mexicana* (Saussure, 1867) (Hymenoptera: Sphecidae), a new neozoon in Austria. Beiträge zur Entomofaunistik 4: 115-116.
