# Supplementary material for: Environmental niche unfilling but limited options for range expansion by active dispersion in an alien cavity-nesting wasp
Source: BMC Ecol. 2018 Sep 20;18:36. doi: 10.1186/s12898-018-0193-9 (PMC6148766; doi:10.1186/s12898-018-0193-9)

Additional file 2. Georeferenced records of *Isodontia mexicana* in Europe (invaded range). The suspected year of arrival of the species, based on the year of the first detection, and the year of the last record, for each of the 17 invaded countries is shown.

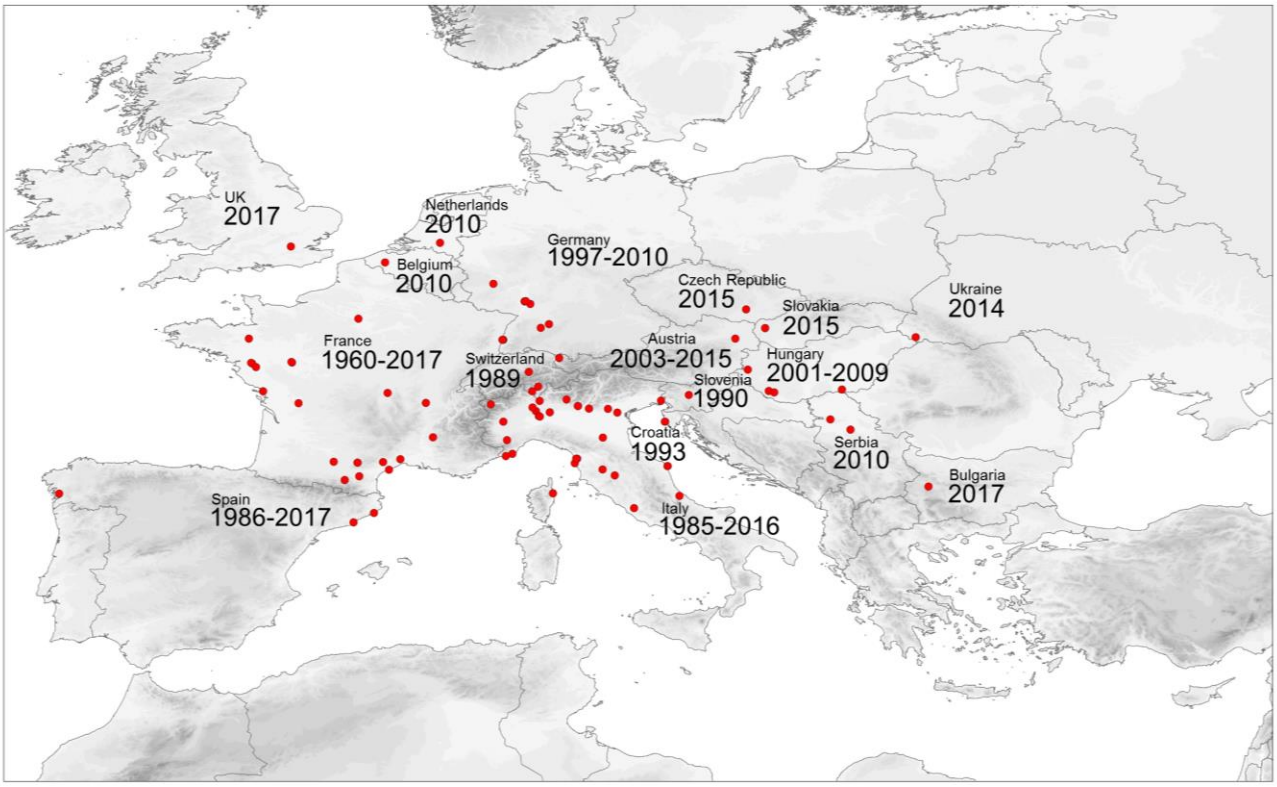

Supplement: Supplementary file 2 — Additional file 2. Georeferenced records of Isodontia mexicana in Europe (invaded range). The suspected year of arrival of the species, based on the year of the first detection, and the year of the last record, for each of the 17 invaded countries is shown. [file 12898_2018_193_MOESM2_ESM.pdf]
