# Supplementary material for: Environmental niche unfilling but limited options for range expansion by active dispersion in an alien cavity-nesting wasp
Source: BMC Ecol. 2018 Sep 20;18:36. doi: 10.1186/s12898-018-0193-9 (PMC6148766; doi:10.1186/s12898-018-0193-9)
Supplement: Supplementary file 3 — Additional file 3. Graphical summary on the known prey use (17 orthopteran genera) by Isodontia mexicana in its native and invaded range. World maps show the rough distribution for each of the 17 genera (“+” indicates occurrence in a given continent; data obtained from GBIF and http://orthoptera.speciesfile.org/HomePage/Orthoptera/HomePage.aspx). Squares indicates the % of the records (n = 47) in which a given genus was found as prey, in both native (white half-square) and invaded (black half-square) range. Picture: I. mexicana female at nest. [file 12898_2018_193_MOESM3_ESM.pdf]

Additional file 3. Graphical summary on the known prey use (17 orthopteran genera) by *Isodontia mexicana* in its native and invaded range. World maps show the rough distribution for each of the 17 genera ("+" indicates occurrence in a given continent; data obtained from GBIF and http://orthoptera.speciesfile.org/HomePage/Orthoptera/HomePage.aspx). Squares indicates the % of the records (n = 47) in which a given genus was found as prey, in both native (white half-square) and invaded (black half-square) range. Picture: *I. mexicana* female at nest.

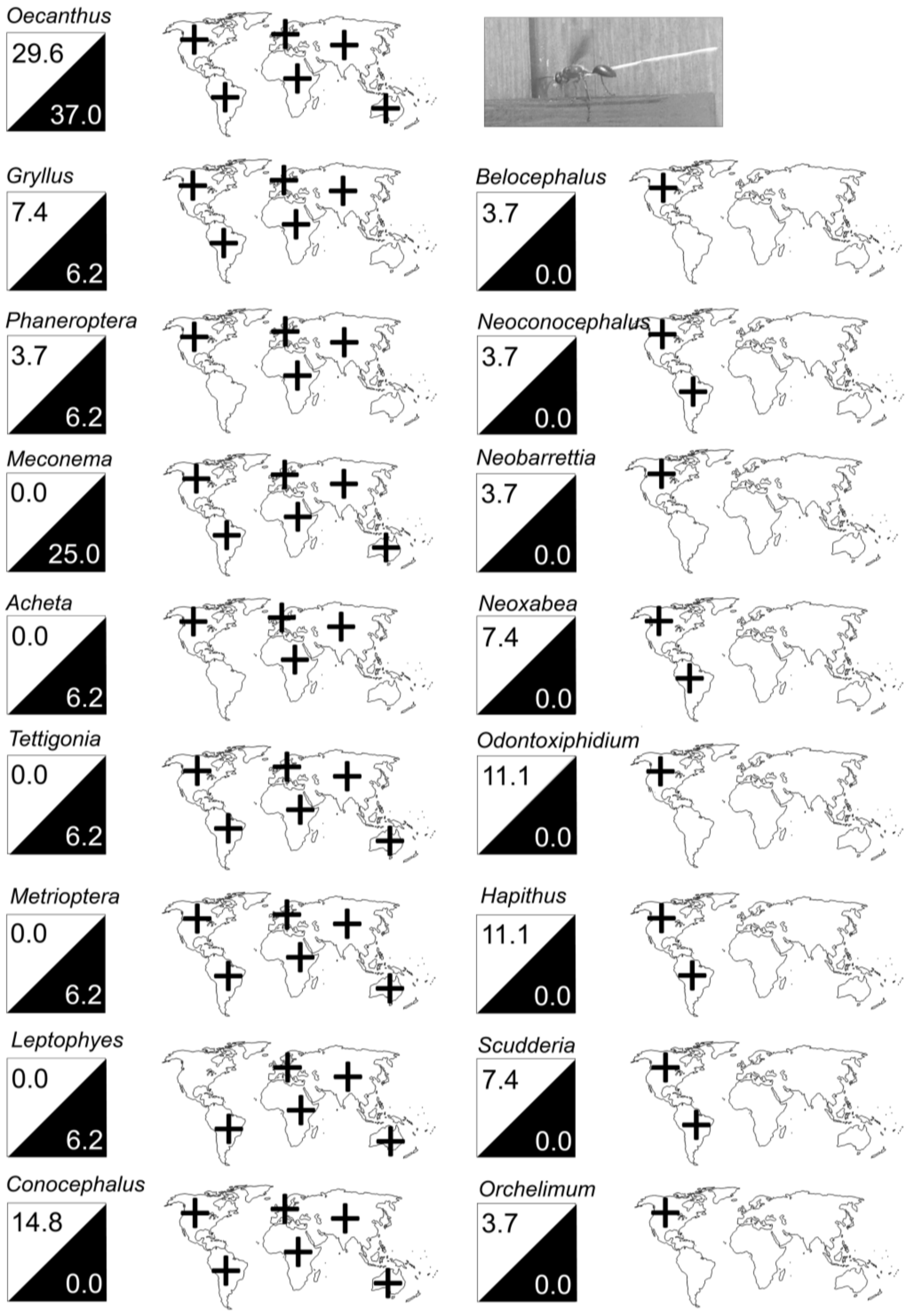

Data on prey use retrieved from the following references:

Bohart RM, Menke AS (1963) A reclassification of the Sphecinae, with a revision of the nearctic species of the tribes Scleiphronini and Sphecini (Hymenoptera:Sphecidae). Univ Cal Pub Entomol 30:91-182

Bohart RM, Menke AS (1976) Sphecid Wasps of the World: a Generic Revision. University of California Press, Berkeley, California

Ercit K (2014) Size and sex of cricket prey predict capture by a sphecid wasp. Ecol Entomol 39:195–202

Fateryga VA, Protsenko YuV, Zhidkov VYu (2014) *Isodontia mexicana* (Hymenoptera, Sphecidae), a new invasive wasp species in the fauna of Ukraine reared from trap-nests in the Crimea. Vestnik Zool 48:185–188

Friebe JG (2015) Der Stahlblaue Grillenjäger *Isodontia mexicana* (Saussure, 1867) (Hymenoptera: Sphecidae) ist in Vorarlberg angekommen. Inatura – Forschung online 24:3S

Entomoland (2003) http://denbourge.free.fr/Insectes_hymenoptera_apocrita_Sphecidae_Isodontia_mexicana.htm. Accessed February 2017.

Jenkins DA, Matthews RW (2004) Cavity-nesting Hymenoptera in disturbed habitats of Georgiaand South Carolina: nest architecture and seasonal occurrence. J Kansas Entomol Soc 77:203–214

Krombein KV (1967) Trap-Nesting Wasps and Bees. Smithsonian Press, Washington, DC.

Krombein KV (1979) Sphecidae. pp. 1575 - 1594. In Krombein, K.V., P.D. Hurd, Jr.. D.R. Smith, and B.D. Burks. cds. Catalog of Hymenoptera in America north of Mexico. Vol.2 (Aculeata). Smithsonian Inst. Press, Washington, D.C.

Lin CS (1966) Bionomics of *Isodontia mexicana*, with a review of generic ethology (Hymenoptera: Sphecidae: Sphecinae.) *Wasmann J Biol* 24:239-247.

Medler JT (1965) Biology of *Isodontia (Murrayella) mexicana* in trap-nests in Wisconsin (Hymenoptera: Sphecidae). Annals Entomological Society of America 58:137–142.

O'Neill KM, O'Neill RP (2003) Sex allocation, nest structure, and prey of *Isodontia mexicana* (Saussure) (Hymenoptera: Sphecidae). J Kansas Entomol Soc 76:447–454

O'Neill KM, O'Neill JF (2009) Prey, Nest Associates, and Sex Ratios of *Isodontia mexicana* (Saussure) (Hymenoptera: Sphecidae) from Two Sites in New York State. *Entomol Am* 115:90-94

Rennwald K (2005) Ist *Isodontia mexicana* (Hymenoptera: Sphecidae) in Deutschland bereits bodenständig? Bembix 19:41–45

Serrano D (2006) Biology, ecology, behavior, parasitoids and response to prescribed fire of cavity nesting Hymenoptera in North Central Florida. Ph. D. Dissertation, University of Florida, Gainesville, Florida, USA. 156 pp.

Tischendorf S, Frommer U, Flügel H-J (2011) Kommentierte Rote Liste der Grabwespen Hessens (Hymenoptera: Crabronidae, Ampulicidae, Sphecidae). Artenliste, Verbreitung,Gefährdung. Hessische Ministerium fürUmwelt, Energie, Landwirtschaft und Verbraucherschutz, Wiesbaden, Germany

Tussac H, Voisin J-F (1989) Observation sur la nidification *d'Isodontia mexicana* (SAUSSURE,1867) en France et en Espagne. Bull Soc Entomol Fr 94:109-111

Vernier R (1995) *Isodontia mexicana* (Sauss.), un Sphecini américain naturalisé en Suisse (Hymenoptera, Sphecidae). Bull Soc Entomol Suisse 68:169-177

Westrich P (1998) Die Grabwespe *Isodontia mexicana* (SAUSSURE 1867) nun auch in Deutschland gefunden (Hymenoptera, Sphecidae). Entomologische Zeitschrift 108: 24-25.
